# Pesticide-Residue Analysis in Soils by the QuEChERS Method: A Review

**DOI:** 10.3390/molecules27134323

**Published:** 2022-07-05

**Authors:** Miguel Ángel González-Curbelo, Diana Angélica Varela-Martínez, Diego Alejandro Riaño-Herrera

**Affiliations:** 1 Departamento de Ciencias Básicas, Facultad de Ingeniería, Universidad EAN, Calle 79 nº 11-45, Bogotá 110221, Colombia; davarela@universidadean.edu.co; 2 Departamento de Ingeniería Ambiental y Energías, Facultad de Ingeniería, Universidad EAN, Calle 79 nº 11-45, Bogotá 110221, Colombia

**Keywords:** green extraction techniques, sample preparation, clean-up, multiresidue analysis, environmentally friendly methods

## Abstract

Pesticides are among the most important contaminants worldwide due to their wide use, persistence, and toxicity. Their presence in soils is not only important from an environmental point of view, but also for food safety issues, since such residues can migrate from soils to food. However, soils are extremely complex matrices, which present a challenge to any analytical chemist, since the extraction of a wide range of compounds with diverse physicochemical properties, such as pesticides, at trace levels is not an easy task. In this context, the QuEChERS method (standing for quick, easy, cheap, effective, rugged, and safe) has become one of the most green and sustainable alternatives in this field due to its inherent advantages, such as fast sample preparation, the minimal use of hazardous reagents and solvents, simplicity, and low cost. This review is aimed at providing a critical revision of the most relevant modifications of the QuEChERS method (including the extraction and clean-up steps of the method) for pesticide-residue analysis in soils.

## 1. Introduction

The current widespread use of herbicides, insecticides, fungicides, or other types of pesticides to effectively protect crops from pests and increase agricultural productivity results in unintended negative environmental effects, especially when good agricultural practices are breached. Soils are directly sprayed with pesticides before sowing and at the stage of germination; pesticides can also reach the soil after their application onto crops, even from long distances, through atmospheric volatilization and deposition processes. Consequently, soils contaminated by pesticide residues can be found, even in remote areas where they have never been used [1]. Because most pesticides do not easily dissipate or are biologically or chemically decomposed, their residues can persist in soils, which places soils among the environmental systems most affected by pollution. In fact, this is one of the most significant significant with converting conventional crops to organic, since conventional agriculture depends on the use of pesticides. In this sense, one of the most complete studies to have been carried out recently found that 83% of the 317 agricultural soils analyzed contained one or more residues and 58% contained a mixture [2]. Since pesticide residues have high levels of acute toxicity and endocrine disruptor effects, even at low concentrations, as well as long half-lives, they can affect soil functions, as well as the safety of subsequent productions. In addition, depending on the absorption capacity of the soil materials (pesticides are more strongly absorbed in soils with high clay or organic matter content than in sandy soils) and other environmental conditions, such as temperature, humidity, and pH, pesticide residues can migrate to other environmental compartments, such as ground or surface water. For this reason, water quality is normally monitored near to agricultural areas. Regarding the potential health risks to humans, they are not only exposed indirectly to pesticide residues through food grown in contaminated soil or products derived from grazing animals, but also by the ingestion/inhalation of soil and dust particles, as well as by dermal contact [3]. Hence, current legislation is increasingly restrictive to protect ecological sustainability and human health, even in developing countries, where there is an increase in the application of methods of food production that adopt the maximum residue levels established by international institutions, such as Codex Alimentarius. This implies the need to continuously develop new methods of analysis to assess these residues at trace levels in a fast, economical, and reliable way. In this sense, the improvement of sample pretreatments to extract these multiresidues strongly adsorbed into complex and heterogeneous soils is a fundamental aspect that requires constant revision.

The standard sample-extraction methods routinely employed for pesticide residues from soil include the Soxhlet extraction (Environmental Protection Agency (EPA) method 3540), automated Soxhlet extraction (EPA method 3541), pressurized liquid extraction (PLE) (EPA method 3545), microwave-assisted extraction (MAE) (EPA method 3546), ultrasonic solvent extraction (USE) (EPA method 3550), and supercritical fluid extraction (SFE) (EPA method 3562), as well as solid-phase extraction (SPE), solid-phase microextraction (SPME), matrix solid-phase dispersion (MSPD), and accelerated solvent extraction (ASE). Because soil matrices usually have a high content of natural organic components, mainly composed of humic substances, lipids, pigments, and fulvic acids, the matrix effects from the presence of interfering substances in the injection vial that are coextracted with pesticides should be minimized. Therefore, different clean-up steps, such as SPE using alumina (EPA method 3610), Florisil (EPA method 3620), or silica gel (EPA method 3630), as well as gel permeation chromatography (EPA method 3640) and sulfur (EPA method 3660), have also been incorporated into the analytical methods. However, any combination results in multi-stage procedures that use large amounts of toxic organic solvents and time require a large working place, are very tedious, and can discharge substantial waste. Therefore, increasingly environmentally friendly, fast, and simple alternatives are currently being developed to meet new analysis needs and to observe the principles of green chemistry.

**Figure 1:**
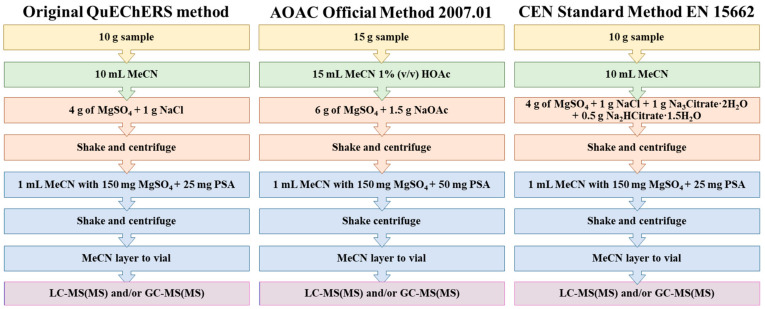
Diagram of the three primary QuEChERS methods based on [5,7,8], respectively.

Alternatively, attention has recently been drawn towards the use of a quick, easy, cheap, effective, rugged, and safe (QuEChERS) method to replace previous, less efficient extraction methods for pesticide determination [4]. It was first presented at the Fourth European Pesticide Residue Workshop (EPRW 2002), published in 2003 by Anastassiades et al. [5], and validated by Lehotay et al. [6]. The QuEChERS method has made it possible to quantify a much broader spectrum of pesticides (even hundreds) from different chemical classes simultaneously in a fast, simple, and cost-effective way while minimizing the amounts of sample and organic solvent used. This environmentally friendly and multiresidue method for the high-throughput routine analysis of pesticides involves only two steps, which minimizes errors: (i) a microscale extraction step with acetonitrile (ACN) based on partitioning via salting-out combined with (ii) a dispersive SPE (d-SPE) using a mix of clean-up sorbents composed of anhydrous MgSO_4_, together with primary secondary amine (PSA) to remove traces of water and matrix interferences (organic acids, fatty acids and sugars), respectively, without large volume transfers or exchanges of solvents, blending, filtration, or evaporation [4]. This allows a single operator to perform multiple extractions simultaneously within a short period of time. The general scheme of the original (unbuffered) QuEChERS method can be observed in Figure 1, which includes the two official buffering-salt methods to increase the recovery of pH-dependent analytes, called the AOAC Official Method 2007.01 [7] and the CEN Standard Method EN 15662 [8]. These methods are called official methods because they were published by the AOAC (Association of Official Analytical Chemists) and the European Committee for Standardization, respectively. Moreover, the QuEChERS method stands out for removing matrix interferences and achieving very accurate results and high sensitivity. Due to all these features, it has evolved into the most popular method for the determination of pesticide residues in soil and related applications [4].

Several reviews have been published in recent years focused on sample preparation procedures for the determination of pesticides in soils, including the QuEChERS method [9,10,11,12,13]. However, to the best of our knowledge, only one of these review articles was critically focused on modifications involving the use of the QuEChERS method for pesticide-residue analysis in soils, and it was published several years ago [11]. Therefore, the aim of this review is to provide an up-to-date critical assessment of the QuEChERS-based methods that have been employed for the analysis of pesticide residues in soils. On this basis, the modifications to the QuEChERS method are thoroughly described as a reference for researchers interested in this subject and in other types of organic contaminant or similar matrices, as well as for private laboratories and state agencies that seek to apply new and cost-effective methods.

## 2. QuEChERS Applications to Pesticide-Residue Analysis in Soils

According to Web of Science, 726 articles have been published featuring the terms “soil”, “pesticide”, and “QuEChERS”, of which only 212 directly focus on the determination of pesticide residues in soils at trace levels by the QuEChERS method and chromatographic techniques coupled to mass spectrometry or other detectors, including the development and validation of analytical methods, as well as monitoring studies. The first application of the QuEChERS method for pesticide-residue analysis in soils was published in 2008 by Lesueur et al. [14]. In that study, the authors compared the QuEChERS method with a new USE, the European Norm DIN 12393, and a PLE method combined with gas chromatography–mass spectrometry (GC–MS) and high-performance liquid chromatography–tandem mass spectrometry (HPLC–MS/MS) in three different types of soil. The QuEChERS method was the most efficient extraction procedure: around 50% of the 24 multiclass pesticides analyzed had recoveries satisfying the 70–120% recovery range and a median recovery of 72.7%. Table 1 [14,15,16,17,18,19,20,21,22,23,24,25,26,27,28,29,30,31,32,33,34,35,36,37,38,39,40,41,42,43,44,45,46,47,48,49,50,51,52,53,54,55,56] summarizes a representative sample of the subsequent studies regarding the QuEChERS extraction approaches for the analysis of a wide range of pesticide residues belonging to different chemical families, such as organochlorine pesticides (OCPs) [15,16,20,25,29,48], organophosphorus pesticides (OPPs) [25], pyrethroid pesticides (PYPs) [25,47], neonicotinoids [35,57], carbamates [53], and triazole [18] and urea [22] derivatives, among others. Depending on the country, the types of pesticides vary due to the characteristic crops of each geographical and climatic zone. In most of these works, HPLC coupled with MS or MS/MS was the technique adopted for the determination of the pesticide residues, followed by GC-MS(MS), because it shows limitations for volatile pesticides, while HPLC allows the separation of the thermolabile and polar residues, as well as showing higher sensitivity. In some cases, ultra-high-performance liquid chromatography (UHPLC) coupled with MS/MS was employed for high throughput, especially when hundreds of pesticides were analyzed simultaneously [49,50]. Less sensitive techniques for pesticide residue analysis in soil samples include HPLC with traditional detectors, such as diode array detectors (DADs) [17,35], fluorescence detectors (FLDs) [53], ultraviolet (UV) [52] and GC with electron-capture detectors (ECDs) [16,25,29,36], and nitrogen phosphorous detectors (NPDs) [36] or flame photometric detectors (FPDs) [25] for OCPs and OPPs, respectively. In this context, Łozowicka et al. [36] studied the extent and variability of the matrix effects of pesticides using GC with different types of detectors (MS/MS and µECD/NPD). In the case of MS/MS detection, the recoveries for almost all the pesticides were in the range of 70–120% with an acceptable relative standard deviation (RSD) of less than 17% while μECD/NPD detection gave recoveries in the range 60–69% with similar RSD values. Unfortunately, the results for both systems of detection remained poor for captan, dichlofluanid, folpet, thiabendazole, and tolylfluanid, with recoveries between 63 and 69%. Nevertheless, it is well known that captan and folpet tend to degrade when they are pesticides are dissolved in ACN solutions [58], which was the extraction solvent. Analogously, Yang et al. [43] clearly observed some interfering compounds in the chromatograms of GC–ECD for the assessment of chloroacetanilide herbicides, which may cause overestimations or even false-positives. Therefore, the GC–MS/MS was more suitable for the analysis of those herbicides. Even though most MS/MS techniques provide high selectivity and sensitivity [59], sample preparation is still crucial. In this sense, the original version [5] and the two official versions [7,8] of the QuEChERS method were developed for the determination of pesticides in fruits and vegetables. This is why different modifications of the QuEChERS method for the extraction of pesticides from soils have been developed. Many of them focus on optimizing the parameters of both the extraction step and the subsequent clean-up step. These improvements have been made with the aim of obtaining better extraction efficiency and providing greater reliability and robustness to the chromatographic system, which is usually sensitive to matrix effects [36].

## 3. The Extraction Step

The original approach, which involves adding anhydrous magnesium sulphate and sodium chloride in the extraction step, has found several applications for the analysis of pesticide residues in soils [18,22,26,28,37,38,48]. Furthermore, many researchers have used extraction liquid–liquid partitioning based on the AOAC Official Method 2007.01, which involves the use of acetic acid (HAc) in can, plus anhydrous MgSO_4_ and NaOAc (relatively strong buffering capacity) [31], and the CEN Standard Method EN 15662 approach, which uses ACN followed by anhydrous MgSO_4_ and NaCl, as well as sodium citrate tribasic dihydrate and sodium citrate dibasic sesquihydrate as the buffer (with a relatively low buffering capacity) [14,19,20,24,27,30,36,44,51]. In an interesting example, Yu et al. [31] compared the original method with the AOAC 2007.01 and EN 15662 official methods for the extraction of 58 multiclass pesticides from soil samples. Concretely, the no-buffer method contained 4 g of MgSO_4_ and 1 g of NaCl, the acetate buffer contained 4 g of MgSO_4_ and 1 g of NaOAc, and the citrate buffer contained 4 g of MgSO_4_, 1 g of NaCl, 1 g of sodium citrate tribasic dihydrate, and 0.5 g of sodium citrate dibasic sesquihydrate. The AOAC QuEChERS version gave the higher average recoveries, between 72% and 121% (RSD < 19%), while the EN buffer method gave slightly lower recoveries (67–123%, RSD < 15%). The recoveries for the no-buffer method were lower than 70% for approximately 30% of all the pesticides. It should be noted that although the original works established specific amounts of reagent, many of these studies were slightly modified to obtain increasingly effective methods for high-organic-matter-content and low-humidity-content soils. Based on the analysis of the works under study, it was determined that the factors with the greatest impact on the extraction of pesticides from soils by the QuEChERS are: (i) the sample mass, (ii) the type and volume of solvent, and iii) the type and amount of extraction salt.

### 3.1. Modifications of the Sample Amount

The amount of sample and even sample size selection play an important role in obtaining the most accurate possible analytical results and high sensitivity. In this context, different sample amounts have been extracted after proper homogenization through mechanical processes, such as grinding and sieving. Methods involving 1 g [53], 2 g [20], 2.5 g [16], 5 g [52], 10 g [51], 15 g [17], and 20 g [33] of soil sample have been developed for pesticide-residue analysis, although most authors opted for 5 or 10 g. In all cases, the amount of sample used can be considered relatively low, which in turn is one of the great inherent benefits of the QuEChERS method. However, it must be considered that the extraction is normally carried out in 50-milliliter centrifuge tubes. Consequently, smaller sample amounts allow good homogenization and better separation of the supernatant because there is more free volume. Unfortunately, the lower the amount of sample, the lower the amount of analyte injected in the chromatographic system, so a proper balance must be found between the amount of sample that provides acceptable recoveries and the required sensitivity. Fernández et al. [24] reduced the sample amount from 10 g to 5 g, achieving a higher mean recovery (104% versus 68%) for the 36 multiclass pesticides analyzed by the CEN Standard Method EN 15662 and GC–MS. Correia-Sá et al. [60] also reduced the sample amount from 10 g to 5 g because no volume of supernatant could be taken, but they added only 3 mL of H_2_O to hydrate the sample and 7 mL of ACN as the extraction solvent, plus 4 g of MgSO_4_, 1 g of NaCl, 1 g of sodium citrate tribasic dihydrate, and 0.5 g of sodium citrate dibasic sesquihydrate. Chen et al. [45], using a 5gram sample amount, obtained recoveries in the range of 72–108%, but the limits of quantification (LOQs) were relatively high, between 80 and 400 µg/kg for the simultaneous determination of 25 multiclass pesticides followed by HPLC–MS/MS. By contrast, Yu et al. [31], applying a 5-gram amount, reached low LOQs, within the range of 0.1–5 µg/kg, for 58 multiclass pesticides by using the AOAC buffer method combined with GC–MS/MS. For smaller sample amounts, Rouvière [20] obtained worse LOQs in the range 6.9–2118 µg/kg using 2 g of sample by the EN citrate buffer method and GC–MS.

### 3.2. Modifications of Water Addition during Extraction

The QuEChERS method was originally developed for matrices with a high water content (above 80%) [5]. Later, it was applied to dry matrices, such as cereal samples, in which a sample rehydration step was implemented by shaking before extraction [61,62,63]. Because soil is a matrix with a low moisture content, the addition of water has also been considered in most pesticide extractions from soil samples. This additional step makes it possible to promote a moisturizing process. In addition, it alters the formation of H-bonds between the functional groups of non-ionic polar pesticides and those containing oxygen and hydroxyl of humic substances to achieve maximum extraction yield and accurate results [64]. However, although the QuEChERS approach recommends that the amount of water added should be the same as the mass of the sample, different ratios of soil to water have been studied with different volumes of water. Yang et al. [43] studied different amounts of water (2, 4, 6, 8, 10, and 15 mL) added to 5 g of soil sample. The results showed that 10 mL of water provided a cleaner extract and an increase in the signal-to-noise ratio (15.0 mL did not improve the results) for the six chloroacetamide herbicides analyzed by GC–MS/MS. Łozowicka et al. [36] tested cold-water dosages of 5, 7.5, and 10 mL with 5 g of soil sample. The use of cold water prevents the degradation of heat-sensitive pesticides that occurs when anhydrous MgSO_4_ is added during extraction. When 10 mL of water were added, better recoveries were obtained for about 40% of the 216 multiclass pesticides compared to 7.5 mL. In the case of 5 mL of water, no supernatant was obtained. Correia-Sá [60] found that the best recoveries for all the tested pesticides were obtained with the hydration step with a ratio of 5 g to 3 mL (recoveries ranged from 77 to 130% versus 20 to 46% without H_2_O addition). By contrast, Acosta-Dacal et al. [50] added water to aliquots of an air-dried soil sample to reach 10, 20, 30, 40, and 50% moisture. As the percentage of moisture increased, the authors did not observe significant differences in the recovery values of the pesticides determined by UHPLC/MS-MS. Instead, the recoveries were worse for many of the pesticides analyzed in GC–MS/MS with the increase in moisture, which was related to the reduction in the matrix load in the sample and, therefore, the sensitivity. These apparently contradictory results confirm the importance of optimizing the hydration step for the successful extraction of pesticides from soils.

### 3.3. Modifications of the Extraction-Solvent Type

As is well known, the selection of an appropriate extraction solvent plays a decisive role in achieving the maximum recovery of pesticides. Several solvents, such as ethyl acetate (EtOAc) [16], MeOH [46], dichloromethane (DCM) [20], or different mixtures [15,35] have been used for multiresidue pesticide analysis in soil samples by the QuEChERS method. However, EtOAc poorly extracts the most highly polar pesticides, MeOH coextracts large amounts of interfering substances from the matrix, and DCM is a highly toxic organochlorine solvent. Instead, ACN is the default extraction solvent used in this method because it efficiently isolates a wide range of polar and nonpolar pesticides while minimizing the amount of coextracted undesirable lipophilic compounds; hence, it provides higher selectivity for pesticide analyses [5]. In this context, Chen et al. [45] compared MeOH, DCM, and ACN as extraction solvents for 25 herbicides, obtaining poor recoveries between 54–108% and 37–110% for the MeOH and DCM, respectively, but acceptable and consistent recoveries in the range of 71–113% when the ACN was used. Similarly, Guan et al. [41] found that ACN gave higher extraction efficiencies than acetone, EtOAc, acetone/hexane, and acetone/DCM for the determination of diniconazole, fipronil, flutriafol, hexaconazole, picoxystrobin, tebuconazole, and triadimenol by UHPLC–MS/MS. ACN was also selected by Chai et al. [25] for the extraction of ten OCPs, eight OPPs, and six PYPs, obtaining satisfactory recoveries in the range of 80–120%, 82–118%, and 87–112%, respectively, with RSD values lower than 11% in all cases. Other ACN-based QuEChERS methods have also been successfully validated for the simultaneous extraction of 216 [36], 225 [49], and 218 [50] pesticides belonging to very diverse chemical families. In addition, ACN is less toxic than DCM, which was one of the most widely used solvents for many years [13], making QuEChERS more environmentally friendly. It should also not be forgotten that ACN can be easily separated from water by adding salt and subsequent centrifugation, which allows the more efficient removal of residual water compared to other solvents [5], and it is highly compatible with GC and HPLC/UHPLC analysis. Thus, the implementation of additional evaporation and reconstitution steps is not necessary. As disadvantages, ACN has a large solvent-expansion volume for GC analysis, and it is expensive. However, ACN is still the most commonly employed extraction solvent in the QuEChERS method for pesticide-residue analysis in soils using relatively small volumes, usually between 5 and 15 mL, with a sample-to-solvent ratio of 1 g per mL [17,22,23,44] or 0.5 g per mL [24,27,38,43,52]. The optimization step has also included yield experiments with acidified ACN. On one hand, HAc has been added, normally at 1%, to form the HAc/NaOAC buffer, which is the basis of the AOAC version, to prevent the degradation of alkali-sensitive pesticides, but it has also been included without the subsequent addition of NaOAc [18,21,25,26,30,34]. On the other hand, formic acid (FA) has also been added to stabilize pesticides that tend to degrade under basic conditions [36,37,49,50,51], even in higher proportions. Xu et al. [40] studied the recoveries of fluopicolide, cyazofamid, and their metabolites with various concentrations of FA (0%, 2%, 2.5%, and 3%), while Acosta-Dacal et al. [50] compared extractions using ACN containing HAc (1%), FA (0.5%, 1% and 2.5%), and no added acid for the analysis of 218 multiclass pesticides. In both works, the addition of FA at 2.5% was the best choice. Combinations of ACN with other solvents, such as EtOAc [15] and DCM [35], have rarely been applied for very volatile pesticides.

### 3.4. Modifications of the Salting-Out Effect

As stated at the beginning of this section, the three main versions of the QuEChERS method, each with its characteristic salts, have been widely applied to extract pesticides from soil samples. However, other combinations of the same salts, or even different salts, have been assayed to promote ACN/water-phase separation during extraction. In this sense, the combination of anhydrous MgSO_4_ and NaCl in 1:1 [39] or 2:1 [32,34,52,53,57] ratios (*w/w*) have also been used as alternatives to the original ratio, and both salts have even been successfully used alone. As an example, García Pinto et al. [16] performed a series of experiments with different combinations of anhydrous MgSO_4_ with and without NaCl for the extraction of chloroform, 1,2-dichlorobenzene, and hexachlorobenzene (HCB). The results showed that there were no significant differences between them, and only anhydrous MgSO_4_ was used in the final method. Nevertheless, it is well known that the use of MgSO_4_ alone can lead to the presence of higher co-extractives [5]. For its part, NaCl alone has been directly used in other works, even without any previous study or optimization, due to its ability to improve the recoveries of polar compounds [23,35,40,43,45]. In the work carried out by Salama et al. [65], the authors used a central composite design to optimize the humidity (4, 5, and 6 mL of water), shaking time (3, 5, and 7 min), and amount of NaCl (1, 1.5 and 2 g) for the extraction of 30 multiclass pesticides. Although the humidity and shaking time had the most significant effects on the selected responses, the amount of NaCl had no significant effect. The most favorable extraction performance was obtained using 6 mL of water, a 7-minite shaking time, and 1 g NaCl. In the case of the citrate and acetate buffers, the salts were mostly added in the same 2:1 and 3:1 ratios (*w/w*), respectively, as in the official methods, but the combination of anhydrous MgSO_4_ and NaOAc has also been added in a 4:1 ratio (*w/w*) [31]. In this last case, this combination was compared with that of the EN and original versions and gave better recoveries in the range of 72–121% versus 67–123% (the recoveries were slightly lower for several pesticides) and lower than 70% for approximately 30% of the 58 pesticides studied, respectively. It is also important to mention the work of Feride et al. [66], which tested the extraction efficiency of different salts (MgSO_4_, NaCl, K_2_CO_3_, Na_2_SO_4_, and NaOAc) for the simultaneous extraction of 42 multiclass pesticides and 23 multiclass industrial chemicals. The higher extraction efficiency was obtained using ACN containing 1% HAc and a combination of MgSO_4_, NaCl, and NaOAc (4:1:1, *w*/*w*). Much less commonly, some authors have applied the QuEChERS method without including extraction salts [41,42,56]. Evidently, these authors did not add water to the soil sample as a hydration step.

## 4. The Clean-Up Step

Soil is an extremely complex matrix that typically requires a clean-up step prior to injection into the chromatographic system to remove undesired coextracted substances and minimize the matrix effect. These substances can act as interferences and negatively affect the reproducibility and sensitivity of the pesticide quantification, as well as increasing the need for equipment maintenance [67,68]. Therefore, in addition to the recovery assessment, the impact on the instrumental performance must also be considered. In this sense, the higher the organic matter content of the soil, the greater the attention that should be paid to the format and sorbent formulations of the clean-up step of the QuEChERS method.

### 4.1. The d-SPE Approach

**Figure 2:**
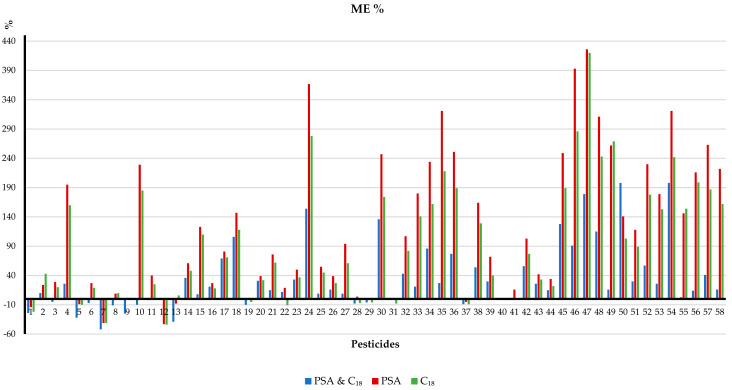
Matrix effects of the comparisons between different combinations of clean-up sorbents in soil samples. When matrix-effect (%) values are 0%, there is no matrix effect. Matrix-effect (%) values between 20% and 20% are mild. Matrix-effect (%) values between −50% and −20% or 20% and 50% are medium. Matrix-effect (%) values below 50% or above 50% are strong. Reprinted from [31], with permission from The Royal Society of Chemistry.

In the first publication of the QuEChERS method, Anastassiades et al. [5] introduced the concept of dSPE as a powerful clean-up procedure to adsorb interferences by adding a small quantity of sorbents into an extract, while the target pesticides remained in the liquid phase. The supernatant was then separated by centrifugation. Therefore, dSPE does not require the use of columns and frits, vacuum manifolds, preconditioning steps, the collection and evaporation of solvent fractions, etc. Consequently, dSPE is a shorter, simpler and more environmentally friendly procedure than conventional SPE. As in the most popular versions, anhydrous MgSO_4_ and PSA have been effectively used during pesticide analysis in soil samples [14,17,19,22,53,54,56]. For example, Słowik-Borowiec et al. [54] recently used 180 mg of MgSO_4_ and 30 mg of PSA per mL of extract for the determination of 94 multiclass pesticides and 13 polycyclic aromatic hydrocarbons in soil samples. The sample extraction was carried out using a modified EN QuEChERS version, and the final instrumental analysis was completed by GC–MS/MS, achieving satisfactory recoveries from 70% to 117% and RSD values in the range of 0.6–15.4%. However, this should not be the default choice because the use of PSA can lead to the hydrolysis of alkali-sensitive pesticides due to its basicity [69]. Consequently, the type and amount of cleaning sorbent have been the factors that have received the most attention for these applications. In this regard, a considerable number of studies have been published on the combination of MgSO_4_ and/or PSA with other common sorbents, such as octadecylsilane (C_18_) [23,24,27,28,31,34,44,51,55] and/or graphitized carbon black (GCB) [41,43,57]. On one hand, since C_18_ is a reversed-phase sorbent which has been particularly effective at removing nonpolar interferences from fatty extracts [4], it would be very useful to clean extracts from soils with high organic matter. In fact, C_18_ has been used with MgSO_4_ only [28,34,51] and even alone [35,42,45]. In a representative study, Yu et al. [31] evaluated the addition of (1) 900 mg of MgSO_4_ + 150 mg of PSA + 150 mg of C_18_, (2) 900 mg of MgSO_4_ + 150 mg of PSA, and (3) 900 mg of MgSO_4_ + 150 mg of C_18_ per 6 mL of supernatant in terms of the matrix effect and recoveries for the determination of 58 multiclass pesticides using the AOAC extraction version and GC–MS/MS. According to Figure 2, although most of the analytes exhibited matrix-enhancement effects, the MgSO_4_ + PSA + C_18_ combination gave lower matrix effects than the other two combinations. The recoveries were in the range between 70% and 120% for all three sets for most of the pesticides. On the other hand, GCB is a planar molecule that has been added to remove pigments (chlorophyll and carotenoids), but its addition should be carefully evaluated because it has a strong affinity for pesticides with planar structures, such as HCB, and can cause low recoveries [43,70]. For example, Chen et al. [45] demonstrated that more than half of the 25 herbicides they analyzed exhibited notable recovery loss when 50 mg of PSA (23–80%) or GCB (23–74%,) per mL of extract were used. Instead, the addition of 50 mg of C_18_ per mL of extract achieved satisfactory recoveries in the range of 72–108%, but the corresponding combinations were not evaluated. By contrast, Yang et al. [43] validated the purification effect of the same sorbents alone and in different ratios. The results showed that higher recoveries, in the range of 87–108%, were obtained for most of the six herbicides by combining PSA/GCB/C_18_ with 75–80% using C_18_, 86–96% using GCB, and 90–103% using PSA. The results in terms of the matrix effect were consistent, with values of −11% to 5%, 18% to −25%, −20% to −25%, and −20% to 13%, respectively.

**Figure 3:**
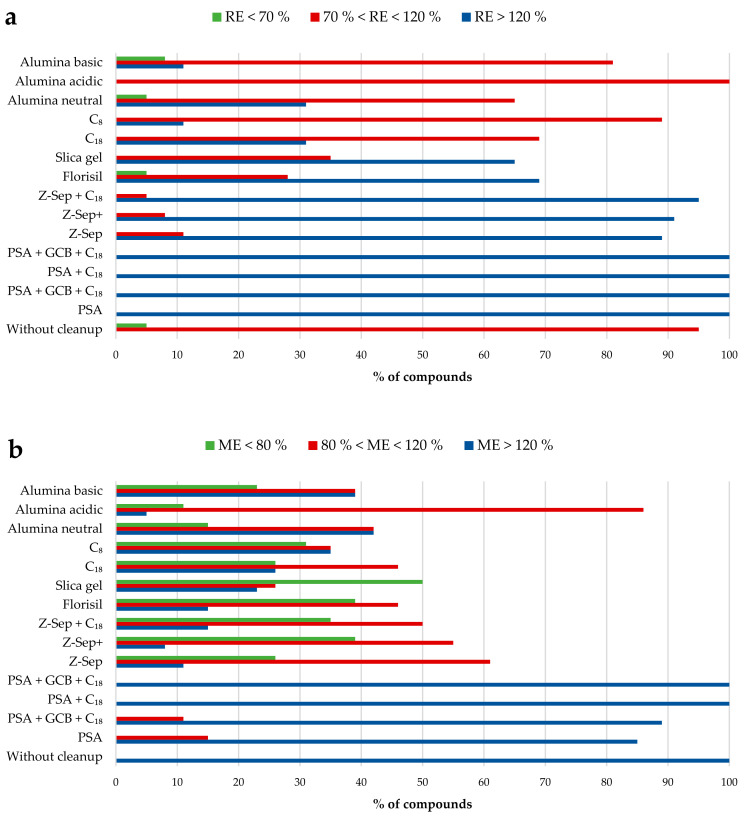
(**a**) Recoveries and (**b**) matrix effects of acid herbicides from various d-SPE sorbents. When matrix-effect (%) values are near to 100%, there is no matrix effect. Matrix-effect (%) values between 80% and 120% are mild. Matrix-effect (%) values below 80% or above 120% are strong. Reprinted from [30], with permission from Elsevier.

Other, less commonly used sorbents for the removal of interfering substances from soil samples are alumina [30], chitosan [26], nanosheets of graphitic carbon nitride (GCN) [32], and Florisil [37,38]. In a related comparative study, Łozowicka et al. [37] evaluated eight clean-up sorbents, namely PSA, GCB, C_18_, alumina, chitosan, Florisil, diatomaceous earth, VERDE, and ChloroFiltr, for the determination of spirotetramat and its four metabolites (β-enol, β -keto, β -mono, and β -glu) in terms of the matrix effect and recoveries. The results showed that the Florisil (200 mg; 6 mL extract) provided the lowest matrix effect and recoveries between 76 and 94%, with RSD < 12%. Analogously, Dong et al. [38] demonstrated that Florisil gave better results in terms of extraction efficiency for the determination of metaldehyde and niclosamide ethanolamine than PSA, GCB, and multi-walled carbon nanotubes (MWCNTs). Nevertheless, Oliveira-Arias et al. [26] found that chitosan or diatomaceous earth achieved better results in terms of extraction efficiency and matrix effect compared to PSA, chitin (50 mg each together with 150 mg of MgSO_4_ per 2 mL of extract), and no clean-up step for the determination of 17 pesticides from rice-paddy soil by HPLC–MS/MS. Furthermore, Guan et al. [41] compared Florisil (100 mg) with PSA (100 mg), C_18_ (100 mg), GCB (100 mg), PSA + C_18_ (100 mg, 1:1, *w/w*), and PSA + C_18_ + GCB (150 mg, 1:1:1, *w*/*w*/*w*) for the analysis of seven pesticides; slightly higher recoveries were obtained when the mixture of PSA, C_18_, and GCB was used. Subsequently, the amounts of these sorbents were optimized, and the best proportion was a mixture of 50 mg of PSA, 50 mg of C_18_, and 200 mg of GCB, together with 300 mg of MgSO_4_. All the amounts above correspond to a volume of 5 mL of acetonitrile layer. One of the most complete comparative studies regarding the use of different sorbents in the d-SPE step in soil samples was developed by Kaczyński [30]. This study evaluated the purification effect of 14 combinations ((1) 25 mg PSA and 2.5 GCB; (2) 25 mg PSA and 25 mg C_18_; (3) 25 mg PSA + 7.5 mg GCB + 25 mg C_18_; (4) 25 mg PSA; (5) 75 mg Z-Sep; (6) 50 mg Z-Sep+; (7) 20 mg Z-Sep; 50 mg C_18_; (8) 200 mg Florisil; (9) 200 mg silica gel; (10) 200 mg C_18_; (11) 200 mg C_8_; (12) 200 mg alumina neutral; (13) 200 mg alumina acidic; and (14) 200 mg alumina basic per 2 mL of extract without MgSO_4_ in all cases) for the determination of 26 acid herbicides with UHPLC–MS/MS. As a novelty, the authors tested the use of Z-Sep and Z-Sep+ sorbents based on zirconium dioxide, which have been used for commodities containing high amounts of fat [71,72]. However, as can be seen in Figure 3, the use of acidic alumina gave recoveries in an acceptable 70–120% range for all the pesticides (Figure 3a), and the matrix effects were either not significant or mild for the highest number of pesticides (Figure 3b). Acosta-Dacal et al. [50] also tested, for the first time in soil, another new sorbent, called Enhanced Matrix Removal-Lipid (EMR-lipid), specifically designed for high-fat matrices. However, the recoveries of 218 multiclass pesticides determined in an agricultural soil sample from the Canary Islands (clay loam soil) by UHPLC–MS/MS and GC–MS/MS were not improved. In fact, none of the other sorbents evaluated (PSA, C_18_, and GCB) improved; therefore, a one-step QuEChERS-based method without clean-up was selected. 

### 4.2. Other Clean-Up Approaches

Conventional SPE is one of the most commonly used alternatives to d-SPE for different applications despite its operational shortcomings, including the packaging of higher amounts of sorbents in order to obtain good clean-up effects. In the case of soil samples, a SPE method (1000 mg Florisil; 6 mL) was compared with a d-SPE method (150 MgSO_4_, 50 mg PSA and 50 mg C_18_; 1 mL) by Di et al. [29]. The recoveries of all the 10 OCPs analyzed were in the range of 95–115%, with RSD values lower than 5% for the Florisil–SPE cartridge, but lower for the d-SPE approach (31–87%, RSD < 10%). Ma et al. [47] also found slightly better recoveries using an SPE column packed with Florisil (94–99%) compared to a SPE column filled with a mix of MgSO_4_, PSA, and GCB (83–100%) for the analysis of six pesticides. In work developed by Sun et al. [33], the authors compared two different SPE cartridges (HLB and C_18_) to quantify benzobicyclon in soil and sediment samples, and the HLB cartridge showed a slightly better purification effect than the C_18_ cartridge.

In addition to the above-mentioned commercial sorbents, magnetic nanoparticles (MNPs) have been synthetized in the laboratory to selectively remove interference from soil samples. MNPs are also directly introduced in the extract and, once appropriately dispersed, they can easily be separated from it using an external magnet without additional centrifugation. Next, the analytes are eluted with an appropriate solvent. In this relatively novel approach, named magnetic d-SPE, bare magnetite (Fe_3_O_4_) is the most widely used MNP for a number of applications in pesticide-residue analysis, but its selectivity is relatively poor. In the study by Hubetska et al. [48], Fe_3_O_4_@Triton was compared with C_18_, GCB, and Fe_3_O_4_ for the determination of 16 OCPs in avocado and strawberry samples. The nonionic surfactant, Triton X-100, was used as a precursor for the synthesis of the functionalized MNPs because it contains several functional complexes that can selectively bind to pesticides. The use of Fe_3_O_4_@Triton gave higher clean-up efficiency and recoveries than the addition of C_18_ and GCB in the d-SPE format and Fe_3_O_4_ in the magnetic d-SPE format. The use of Fe_3_O_4_@Triton was subsequently validated in soil samples, achieving good recoveries between 65 and 103%.

Disposable pipette extraction (DPX) is a practical SPE method that uses disposable pipette tips, in which the sorbent is contained. The sample extract is then aspirated and thoroughly mixed in a dynamic dispersive manner to achieve rapid equilibration. Consequently, undesirable compounds are concentrated on the sorbent and a clean extract is dispensed directly, without centrifugation. In a key study, Fernández et al. [24] compared, for the first time, the DPX and d-SPE procedures in soil samples using a composition of MgSO_4_, PSA, and C_18_ in both cases. The results demonstrated that there was no significant difference for the two clean-up procedures in terms of recoveries for 36 multiclass pesticides in two types of soil (agricultural and organic) by GC–MS/MS. Another study reported the use of a glass Pasteur pipette packed with 200 mg of PSA for OPPs and 200 mg of silica gel for OCPs and PYPs in mineral and peat soils. For d-SPE using 25 mg PSA, the recoveries were in the range of 20–81%. By contrast, acceptable recoveries between 80 and 120% were obtained for all the pesticides in both soils using the DPX alternative [25]. Considering that the DPX procedure provides faster extraction times and is easy to perform, it is an alternative that should be considered for future applications. However, DPX provides poor filtration due to its screen mesh and does not transfer volumes ideally.

### 4.3. No-Clean-Up Approaches

As seen in the work published by Acosta-Dacal et al. [50], the clean-up step can be omitted to make the QuEChERS method less expensive, simpler, and faster, without compromising the analytical performance. This is not the case for most of the soil samples reported in the literature, but some other cases have been described in soils with relatively simple matrix compositions, mostly combined with MS/MS detection or selective detectors. Rouvière et al. [20] purified an extract from peat samples after extraction with ACN or DCM by d-SPE on PSA, but the recoveries of the 34 OCPs analyzed by GC–MS showed that the clean-up step was not necessary. Similarly, Caldas et al. [18] studied the influence of PSA and C_18_ during the analysis of clomazone, fipronil, tebuconazole, propiconazole, and azoxystrobin by HPLC-MS/MS, but these d-SPE sorbents did not have a significant influence on the recovery of the pesticides. Łozowicka [36] compared the EN QuEChERS version with and without the d-SPE step followed by GC–MS/MS and GC–μECD/NPD to determine 216 pesticides. Different combinations of PSA, C_18_, and GCB together with MgSO_4_ were tested, but the use of these sorbents did not have a significant influence on the recoveries or the matrix effect. Subsequently, the QuEChERS procedure without the d-SPE step was successfully validated and applied to the analysis of 263 soil samples. It should be noted that the authors placed the sample extracts in the freezer at −60 °C for 30 min right after the extraction, which is a clean-up process. In fact, this is the simplest method for fat removal from extracts [73,74]. However, it is clearly time-consuming and complicates the procedure.

## 5. Comparison of the QuEChERS Method with Other Extraction Methods

**Figure 4:**
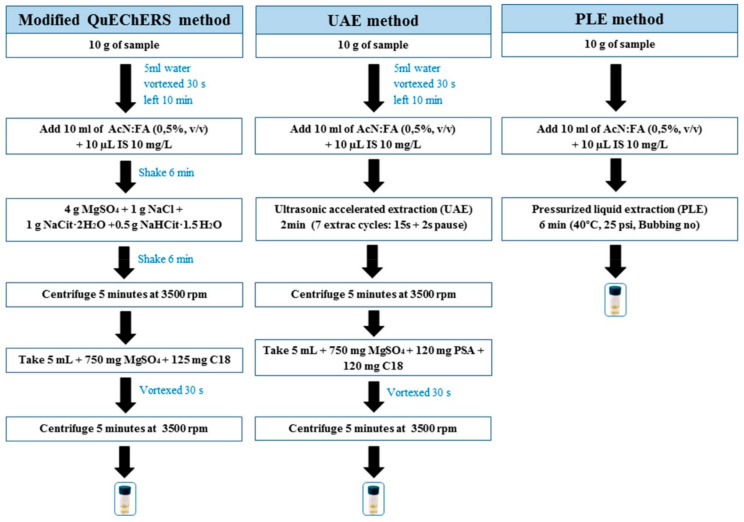
Diagram of the three methods used for the agricultural soil sample extraction. Reprinted from [51] with permission from Elsevier.

The analytical performance of the QuEChERS method for the analysis of pesticide residues in soils has been compared with other extraction methods, such as accelerated solvent extraction (ASE) [20,29], MAE [29], PLE [14,21,27,51,55], solid–liquid extraction (SLE) [21,44], Soxhlet extraction [44], and USE [14,21,29,51]. Although ASE, MAE, and USE were developed as more practical, faster, and more environmentally friendly procedures than the Soxhlet method, the QuEChERS method has since become the first choice of analytical chemists due to its high-throughput performance and easy modification according to the analytical needs of specific combinations of analytes and matrices. Ðurović-Pejčev et al. [44], for instance, reported that the QuEChERS method provided higher extraction efficiency than traditional SLE and Soxhlet for most of the twelve pesticides belonging to the eight chemical groups analyzed in soil samples by GC–MS. Concretely, the recoveries applying the QuEChERS method were in the range of 54–103%, while the recoveries using SLE and Soxhlet were 40–91% and 12–92%, respectively. In turn, Rouvière [20] compared a previously optimized QuEChERS version using DCM as the extraction solvent with an ASE procedure for the analysis of 34 OCPs in soil by GC–MS. The average recovery varied between 60% and 100% when using QuEChERS, which, according to the authors, it proved to be simpler and faster. The ASE was a more tedious procedure and provided worse recoveries for most of the pesticides, ranging from 42% to 85%. In the study developed by García-Valverde [51], a modified QuEChERS version, when compared with an ultrasonic cylindrical probe and PLE combined with UHPLC–MS/MS (see Figure 4), proved to be more efficient (with higher recoveries with up to 12 samples by run) in the determination of 30 organic contaminants of emerging concern, including 13 multiclass pesticides, in agricultural soils, with LOQ < 0.1 ng/g in most cases. Homazava et al. [27] compared the performance, extraction efficiency, and matrix effect of a modified QuEChERS method with PLE followed by UHPLC–MS/MS for the analysis of 25 pesticides. QuEChERS was shown to be less time-consuming and demonstrated a higher sample throughput; recoveries between 79% and 113%, with RSDs of 1.0–12.2%, were obtained. By contrast, PLE extraction only reached recoveries between 65% and 122%, with RSDs of 1.7–23.4%. Moreover, the QuEChERS extracts were clearer and lower matrix effects were obtained (−54.5–7.0% versus −71.7–113.4%). Di et al. [29], for instance, compared the extraction efficiency of QuEChERS, MAE, ASE, and USE procedures combined with GC–ECD and GC–MS/MS for the determination of 10 OCPs. The QuEChERS and MAE procedures were found to achieve recoveries in the ranges of 78–124% (except for o,p’-DDD, with 57%) and 95–115%, respectively, while ASE and USE provided lower recoveries (47–118% and 44–128%, respectively). The authors highlighted the use of purging with nitrogen, in the case of ASE, and the application of ultrasounds, in the case of USE, as possible reasons, particularly for volatile pesticides. Despite the good results of the QuEChERS method, it yielded slightly higher RSD values compared to MAE. Hence, MAE was selected for the further analysis of real soil samples. This shows that, although the QuEChERS method covers a broader scope of pesticides in diverse sample types, providing higher recoveries and better analytical performance than traditional extraction procedures in most cases, for specific applications, there may be more appropriate methodologies. However, the QuEChERS method will continue to be one of the best options for the analysis of pesticide residues in soil.

## 6. Conclusions and Future Trends

The three primary QuEChERS versions have been successfully applied for the simultaneously analysis of multiclass pesticides residues in soil samples due to their short operation time, simplicity, and low cost. The QuEChERS method is also aligned with green chemistry because it decreases the need for toxic solvents and reagents and generates much less waste. In addition, it has been easily adapted to a wide variety of pesticide/soil combinations to yield higher and more robust recovery rates. In this sense, ACN is the principal extraction solvent choice, even when compared to other organic solvents commonly used in this field, for the extraction of soil samples of just 5 or 10 g previously hydrated with similar amounts of water. Furthermore, ACN has been modified by adding HAc or FA as a preventive measure in many cases. Partitioning has been mostly achieved using the characteristic salts of the three main versions. In this sense, the use of a mild citrate buffer is another of the most commonly used default measures. Regarding the clean-up step, MgSO_4_ and PSA are the most commonly used sorbents, whereas C_18_ and GCB have been used for soils with high content of organic matter and pigments, respectively. The use of traditional sorbents, such as Florisil in d-SPE and SPE formats, as well as nanotechnology-based sorbents, such as MNPs, has also been shown to be effective for cleaning purposes. Finally, DPX is a faster alternative to d-SPE and SPE that has hardly been used for soil samples, but for soils with low organic load, the clean-up step might not be necessary.

The QuEChERS method was originally applied to fruits and vegetables and, since then, most of the significant advances have been developed for these matrices before any others. This is the case of MWCNTs, Z-Sep, Z-Sep+, and EMR-lipid, as well as approaches such as magnetic d-SPE and DPX. Therefore, we should consider innovations for the analysis of pesticides and other organic analytes in fruits and vegetables. As an example, the first author of this review and the father of the QuEChERS method developed and validated a new version that uses ammonium formate instead of MgSO_4_, NaCl, NaOAc, or citrate salts to induce phase separation and extraction [75]. Ammonium salts are more volatile, which prevents their deposition as solids in the GC inlet and in the MS ion source, which in turn increases equipment performance and minimizes the need for maintenance and liner replacement. Moreover, ammonium ions can enhance the formation of ammonium adducts instead of undesirable sodium adducts. As in the two official versions of the QuEChERS method, the addition of formic acid achieves suitable buffering. In fact, the performance of the ammonium formate version is similar to that of the QuEChERS AOAC Official Method 2007.01. Therefore, this is an alternative that could be adopted to improve the compatibility between the extraction of pesticides from soils using the QuEChERS method and MS detection. As a better alternative to DPX, filter-vial d-SPE was developed soon afterwards to quickly and conveniently clean and filter extracts in autosampler vials [76]. This approach eliminates centrifugation by combining d-SPE with in-vial filtration. QuEChERS automation is another trend that has gained strength in recent years, making QuEChERS an even faster approach to the analysis of large numbers of samples. Lehotay et al. [77] applied an automated mini-cartridge SPE cleanup combined with low-pressure (LP)GC–MS/MS to yield high-throughput capabilities and to reduce pesticide degradation in long instrumental sequences. Miniaturization is another feature that could be enhanced in the QuEChERS method for the analysis of pesticides in soil samples. Furthermore, the use of non-toxic extraction solvents, such as ionic liquids and deep eutectic solvents, would significantly reduce its waste disposal and costs. A much more recent method is the so-called QuEChERSER, which is an efficient and robust evolution that covers a wider polarity range than the QuEChERS method [78]. QuEChERSER relies on automation and miniaturization simultaneously, employing 1–5-gram samples extracted with 5 mL/g 4:1 (*v*/*v*) ACN-water solution and 1 g per g sample of 4/1 (*w*/*w*) MgSO_4_/NaCl, followed by clean-up using automated instrument-top sample preparation (ITSP or µ-SPE) with 45 mg 20:12:12:1 of MgSO_4_-PSA-C_18_-CarbonX per 300 µL extract for GC (with no additional extraction salts or clean-up step for LC). The QuEChERSER mega-method has already been successfully validated for the analysis of pesticides in fruits and vegetables [79], other pesticides, veterinary drugs, environmental contaminants in bovine muscle [80], catfish muscle [81], tilapia [82], and pesticides in hemp and hemp products [83]. In summary, this is a field in constant evolution that deserves the continuous exploration of new greener, broader-coverage, faster, and cheaper approaches.

## Figures and Tables

**Table 1:** Evolution of the QuEChERS method for the analysis of pesticide residues in soils.

Pesticides	Sample Amount	Water Added	Extraction		Sorbents in the dSPE Step per mL of Extract	Analytical Technique	Recoveries	LOQs	Comments	Reference
			Solvents	Salts						
24 multiclass pesticides	10 g	-	20 mL ACN	4 g MgSO_4_, 1 g NaCl, 1 g sodium citrate tribasic dihydrate and 0.5 g sodium citrate dibasic sesquihydrate	150 mg MgSO_4_ and 25 mg PSA	HPLC-MS/MS and GC-MS	27–121%	0.3–125 µg/kg	The QuEChERS method showed better performance than USE, the European Norm DIN 12393 and PLE	[14]
19 OCPs	5 g	10 mL	10 mL ACN (1% HAc)	4 g MgSO_4_ and 1.7 g NaOAc	-	GC-MS/MS	70–100%	0.1–1.6 µg/kg	The clean-up step was performed by liquid-liquid partitioning with n-hexane	[15]
Chloroform, 1,2-dichlorobenze and HCB	2.5 g	1.5 mL	10 mL EtOAc	4 g MgSO_4_	-	GC-µECD	62–93%	0.4–7.2 µg/kg	EtOAc showed higher extraction efficiency than ACN	[16]
Pyrimorphos	15 g	9 mL	15 mL ACN	6 g MgSO_4_ and 1.5 g NaOAc	150 mg MgSO_4_ and 50 mg PSA	HPLC-DAD	86–96%	50 µg/kg	The clean-up step of the extract was optimized by vortex	[17]
Clomazone, fipronil, tebuconazole, propiconazole and azoxystrobin	10 g	-	10 mL ACN (1% HAc)	4 g MgSO_4_ and 1 g NaCl	-	HPLC-MS/MS	70–118%	10–50 µg/kg	PSA, C_18_ and MgSO_4_ in the d-SPE step did not improve recoveries	[18]
Trifluralin	10 g	-	20 mL ACN	4 g MgSO_4_, 1 g NaCl, 1g sodium citrate tribasic dihydrate and 0.5 g sodium citrate dibasic sesquihydrate	150 mg MgSO_4_ and 25 mg PSA	GC-ECD	87–93%	11 µg/kg	Clean-up and preconcentration steps to change the injection solvent from ACN to EtOAc were incorporated	[19]
34 OCPs	2 g	-	15 mL DCM	4 g MgSO_4_, 1g NaCl, 1g sodium citrate tribasic dihydrate and 0.5 g sodium citrate dibasic sesquihydrate	-	GC-MS	60–100% for almost all pesticides	58–2708 µg/kg	The QuEChERS method showed better performance than ASE. DCM showed higher extraction efficiency than ACN	[20]
Nicotine, sabadine, veratridine, rotenone, azadirachtin, cevadine, deguelin, spynosad D, pyrethrins and piperonyl butoxide	5 g	2.5 mL	5 mL ACN (1% HAc)	4 g MgSO_4_, 4 g NaCl, 1g sodium citrate dihydrate and 0.5 g sodium citrate dibasic sesquihydrate	-	UHPLC-MS/MS	70–120% for almost all pesticides	4–10 µg/kg	The QuEChERS method showed better performance than SLE, SLE-USE and PLE	[21]
Diafenthiuron	10 g	2 mL	10 mL ACN	4 g MgSO_4_ and 1 g NaCl	150 mg MgSO_4_ and 50 mg PSA	HPLC-MS	74–100%	1 µg/kg	USE improved extraction efficiency	[22]
Benazolin-ethyl and quizalofop-p-ethyl	10 g	5 mL	10 mL ACN	3 g NaCl	200 mg PSA and 50 mg C_18_	HPLC-MS/MS	74–110%	5 µg/kg	GCB gave lower recoveries for quizalofop-p-ethyl and benazolin-ethyl	[23]
36 multiclass pesticides	10 g	3 mL	10 mL ACN	4 g MgSO_4_, 1 g NaCl, 1g sodium citrate tribasic dihydrate and 0.5 g sodium citrate dibasic sesquihydrate	d-SPE: 150 mg MgSO_4_, 150 mg PSA and 50 mg C_18_; DPX: 150 mg MgSO_4_, 50 mg PSA and 50 mg	GC-MS/MS	70–120% for almost all pesticides	10 µg/kg	There was no significant difference between d-SPE and DPX in term of recoveries	[24]
10 OPPs, 8 OCPs and 6 PYPs	10 g		15 mL ACN (1% HAc)	6 g MgSO_4_ and 1.5 g NaCl	-	GC-FPD and GC-ECD	80–120%	2–5 µg/kg	0.2 g PSA for OPPs and 0.2 g silica gel format for OCPs and PYPs, both in DPX format using Pasteur pipettes	[25]
17 multiclass pesticides	10 g	-	10 mL ACN (1% HAc)	4 g MgSO_4_ and 1 g NaCl	75 mg MgSO_4_ and 25 mg chitosan	HPLC-MS/MS	70–120% for almost all pesticides	0.1–100 µg/kg	Chitosan was more efficient than PSA, Chitin, and diatomaceous earth for clean-up purposes	[26]
25 multiclass pesticides	5 g	5 mL	10 mL ACN	4 g MgSO_4_, 1g NaCl, 1g sodium citrate tribasic dihydrate and 0.5 g sodium citrate dibasic sesquihydrate	180 mg MgSO_4_, 30 mg PSA and 30 mg C_18_	UHPLC-MS/MS	74–111% for almost all pesticides	0.2–2.5 µg/kg	The QuEChERS method showed better performance than PLE	[27]
Bentazone, atrazine, carbamazepine, phenytoin, and its metabolites 5-(p-hydroxyphenyl-) and 5-phenylhydantoin	5 g	-	10 mL ACN: H_2_O (70:30, *v*/*v*) (5% HAc)	4 g MgSO_4_ and 1 g NaCl	12.5 mg MgSO_4_ and 6.25 mg C_18_	HPLC-UV	83–113%	10 µg/kg	C_18_ showed higher clean-up performance than PSA	[28]
10 OCPs	5 g	3 mL	7 mL ACN	6 g MgSO_4_, 1.5 g NaCl, 1.5 g sodium citrate tribasic dihydrate and 0.75 g sodium citrate dibasic sesquihydrate	-	GC-ECD and GC-MS/MS	57–124%	1–3.6 µg/kg	The QuEChERS and MAE methods showed better performance than ASE and USE, but QuEChERS yielded slightly higher RSD values compared to MAE. Florisil in SPE format showed better clean-up efficiency than a mix of MgSO_4_, PSA and C_18_ in d-SPE format	[29]
26 multiclass pesticides	5 g	10 mL	10 mL ACN (1% HAc)	4 g MgSO_4_, 1 g NaCl, 1 g sodium citrate tribasic dihydrate and 0.5 g sodium citrate dibasic sesquihydrate	100 mg acidic alumina	UHPLC-MS/MS	70–114%	1 µg/kg	Acidic alumina showed better performance compared to 14 combinations of sorbents including PSA, GCB, C_18_, Florisil, silica gel, Z-SEP, and Z-SEP+	[30]
58 multiclass pesticides	5 g	10 mL	10 mL ACN (1% HAc)	4 g MgSO_4_ and 1 g NaOAc	150 mg MgSO_4_, 25 mg PSA and 25 mg C_18_	GC-MS/MS	69–119%	0.1–5 µg/kg	The AOAC QuEChERS version showed better performance than the EN QuEChERS version	[31]
Florasulam, carfentrazone-ethyl, fluroxypyr-meptyl and fluroxypyr	5 g	2 mL	10 mL ACN (1% HAc)	2 g MgSO_4_ and 1 g NaCl	10 mg GCN	HPLC-MS/MS	80–110%	2.4–6 µg/kg	GCN showed higher clean-up performance than C_18_	[32]
Benzobicyclon	20 g	20 mL	40 mL ACN (1% FA)	8 g MgSO_4_ and 2 g NaCl	-	UPLC-MS/MS	64–76%	0.3–2.2 µg/kg	ACN showed higher extraction efficiency than EtOAc. HLB showed higher clean-up performance than C_18_ in SPE format	[33]
Furon, mesotrione, fluroxypyr-mepty and fluroxypyr	5 g	2 mL	10 mL ACN (1% HAc)	2 g MgSO_4_ and 1 g NaCl	200 mg MgSO_4_ and 25 mg C_18_	**1** **HPLC-MS/MS**	80–110%	2.4–6 µg/kg	GCB and PSA were not necessary because the soil had no pigments	[34]
Acetamiprid, imidacloprid, nitenpyram, flonicamid thiacloprid and 6-chloronicotinic acid	10 g	-	25 mL ACN: DCM (1:2, *v*/*v*)	5 g NaCl	400 mg C_18_ for the upper supernatant layer	HPLC-DAD	65–100%	48–246 µg/kg	ACN: DCM (1:2, *v*/*v*) showed higher extraction efficiency than ACN, acetone, EtOAc and ACN: DCM (2:1, *v*/*v*). C_18_ showed higher clean-up performance than PSA	[35]
216 multiclass pesticides	5 g	10 mL	10 mL ACN (1% FA)	4 g MgSO_4_, 1 g NaCl, 1 g sodium citrate tribasic dihydrate and 0.5 g sodium citrate dibasic sesquihydrate	-	GC-MS/MS and GC-μECD/NPD	71–120%	5–10 µg/kg	A clean-up step with different combinations of MgSO_4_, PSA, C_18_ and GCB gave lower recoveries	[36]
Spirotetramat and its four metabolites (β-enol, β-keto, β-mono and β-glu)	5 g	-	10 mL ACN (1% FA)	4 g MgSO_4_ and 1 g NaCl	33 mg Florisil	HPLC-MS/MS	76–94%	1 µg/kg	Florisil showed higher clean-up efficiency than neutral alumina, GCB, PSA, C_18_, diatomaceous earth, VERDE, ChloroFiltr and Chitosan	[37]
Metaldehyde and niclosamide ethanolamine	5 g	-	10 mL ACN	4 g MgSO_4_ and 1 g NaCl	150 mg MgSO_4_ and 50 mg Florisil	HPLC-MS/MS	90–101%	10–200 µg/kg	ACN showed higher extraction efficiency than DCM and EtOAc. Florisil showed higher clean-up efficiency than PSA, GCB, and MWCNTs	[38]
Dioctyl diethylenetriamine acetate	10 g	5 mL	20 mL ACN	**2**→**5 g MgSO_4_ and 5 g NaCl**	-	HPLC-MS/MS	86–97%	10 µg/kg	ACN: H_2_O (4:1, *v*/*v*) showed higher extraction efficiency than MeOH: H_2_O (4:1, *v*/*v*)	[39]
Fluopicolide, cyazofamid and their metabolites (M-01, M-02 and 4-chloro-5-p-tolylimidazole-2-carbonitrile)	10 g	10 mL	ACN 10 mL (2.5% FA)	6 g NaCl	100 mg MgSO_4_	HPLC-MS/MS	71–107%	50 µg/kg	ACN (2.5% FA) showed higher extraction efficiency than ACN	[40]
Hexaconazole, flutriafol, triadimenol, tebuconazole, diniconazole, fipronil and picoxys-trobin	5 g	-	20 mL ACN	-	60 mg MgSO_4_, 10 mg PSA, 10 mg C_18_ and 40 mg GCB	UHPLC-MS/MS	69–106%	0.03–0.25 µg/kg	USE for 20 min improved the extraction efficiency	[41]
Polyoxin B	5 g	-	5 mL H_2_O (1% FA)	-	13 mg C_18_	HPLC-MS/MS	83–112%	3 µg/kg	H_2_O (1% FA) showed higher extraction efficiency than H_2_O: MeOH (1:1), H_2_O and H_2_O (1% NH_3_)	[42]
Acetochlor, alachlor, metolachlor, metazachlor, butachlor and pretilachlor	5 g	10 mL	**3→10 mL ACN**	4 g NaCl	50 mg MgSO_4_, 25 mg PSA, 25 mg C_18_ and 5 mg GCB	GC-MS/MS	87–108%	0.8–2.2 µg/kg	There was no significant difference between ACN and ACN (1% disodium hydrogen citrate sesquihydrate) in terms of recoveries	[43]
12 multiclass pesticides	10 g	20 mL	10 mL ACN	4 g MgSO_4_, 1 g NaCl, 1g sodium citrate tribasic dihydrate and 0.5 g sodium citrate dibasic sesquihydrate	130 mg MgSO_4_, 21 mg PSA and 21 mg C_18_	GC-MS	54–103%	6–21 µg/kg	The QuEChERS method showed better performance than SLE and Soxhlet extraction	[44]
25 multiclass pesticides	5 g	-	20 mL ACN	2 g NaCl	50 mg C_18_	HPLC-MS/MS	72–108%	80–400 µg/kg	ACN showed higher extraction efficiency than MeOH and DCM. C_18_ showed higher clean-up efficiency than PSA and GCB	[45]
Dimethyl disulfide	10 g	5 mL	15 mL MeOH	-	-	GC-MS	85–98%	1 µg/kg	A simplified QuEChERS method without extraction salts showed better performance than the original QuEChERS extraction	[46]
Bifenthrin, chlorfenapyr, λ-cyhalothrin, pyridaben, pyrimethanil, and pyriproxyfen	5 g	-	10 mL ACN (1% HAc)	-	-	GC-MS	86–100%	0.5–2.4 µg/kg	Florisil showed higher clean-up efficiency than a mix of MgSO4, PSA and GCB both in SPE format.	[47]
16 OCPs	5 g	8 mL	8 mL ACN	4 g MgSO_4_ and 1 g NaCl	25 mg Fe_3_O_4_@Triton	GC-MS	65–103%	0.3–5.5 µg/kg	Fe_3_O_4_@Triton showed higher clean-up efficiency than C_18_, GCB and Fe_3_O_4_ in avocado and strawberry, and later this was then validated in soil	[48]
225 multiclass pesticides	10 g	-	10 mL ACN (2.5% FA)	6 g MgSO_4_ and 1.5 g NaOAc	-	UHPLC-MS/MS and GC-MS/MS	70–120 for more than 87% pesticides	1–5 µg/kg	-	[49]
218 multiclass pesticides	10 g	-	10 mL ACN (2.5% FA)	6 g MgSO_4_ and 1.5 g NaOAc	-	UHPLC-MS/MS and GC-MS/MS	70–120%	5–20 µg/kg	The AOAC QuEChERS method showed better performance than the EN QuEChERS version. PSA, C_18_, GCB or EMR- lipid in d-SPE did not improve recoveries	[50]
13 multiclass pesticides	10 g	5 mL	10 mL ACN (0.5% FA)	4 g MgSO_4_, 1 g NaCl, 1g sodium citrate tribasic dihydrate and 0.5 g sodium citrate dibasic sesquihydrate	150 mg MgSO_4_ and 25 mg C_18_	HPLC-MS/MS	70–93%	0.05 µg/kg	The QuEChERS method showed better performance than ultrasonic cylindrical probe and PLE. ACN (0.5% FA) showed higher extraction efficiency than MeOH. PSA did not improve recoveries	[51]
Isocycloseram	5 g	5 mL	10 mL ACN	1 g MgSO_4_ and 0.5 g NaCl	-	HPLC-UV	91–109%	7.3–24 µg/kg	ACN showed higher extraction efficiency than DCM, MeOH, EtOAc and petroleum eter	[52]
Atrazine, desethylatrazin, desisopropylatrazine, carbaryl, carbendazim and diuron	1 g	4 mL	2 mL ACN	1 g MgSO_4_ and 0.5 g NaCl	66 mg MgSO_4_ and 16 mg PSA	HPLC-DAD/FLD	74–108%	5–15 µg/kg	PSA showed higher d-SPE efficiency than Florisil	[53]
94 multiclass pesticides	5 g	10 mL	10 mL acetone:n-hexano (1:4, *v*/*v*)	4 g MgSO_4_, 1 g NaCl, 1g sodium citrate tribasic dihydrate and 0.5 g sodium citrate dibasic sesquihydrate	180 mg MgSO_4_ and 30 mg PSA	GC-MS/MS	70–117%	5–14 µg/kg	Acetone:n-hexane (1:4, *v*/*v*) showed higher extraction efficiency than ACN	[54]
31 multiclass pesticides	2.5 g	6 mL (EDTA 0.1 M)	5 mL ACN	4 g MgSO_4_, 1 g NaCl, 1g sodium citrate tribasic dihydrate and 0.5 g sodium citrate dibasic sesquihydrate	150 mg MgSO_4,_ 25 mg C_18_ and 25 mg PSA	UPLC MS/MS	55–118%	0.01–5.5 µg/kg	The QuEChERS method showed better performance than PLE	[55]
Pyraclostrobin	5 g		10 mL ACN	-	150 mg MgSO_4_ and 50 mg PSA	HPLC-MS/MS	97–102%	0.2 µg/kg	ACN showed higher extraction efficiency than ACN (1% HAc), ACN (0.1% HAc), ACN (1% FA), ACN (0.1% FA) and ACN (1% NH_3_). PSA showed higher clean-up efficiency than C_18_, Florisil, PSA+C_18_ and GCB	[56]

ACN: Acetonitrile; AOAC: Association of Official Analytical Chemists; ASE: Accelerated solvent extraction; C_18_: Octadecylsilane; DAD: Diode array detector; DCM: Dichloromethane; DPX: Disposable pipette extraction; d-SPE: Dispersive solid-phase extraction; ECD: Electron capture detector; EMR-lipid: Enhanced Matrix Removal-lipid; EtOAc: Ethyl acetate; FA: Formic acid; GC: Gas chromatography; FLD: Fluorescence detector; FPD: Flame photometric detector; GCB: Graphitized carbon black; GCN: Graphitic carbon nitride; HAc: Acetic acid; HCB: Hexachlorobenzene; HPLC: High-performance liquid chromatography; LOQ: Limit of quantification; MAE: Microwave-assisted extraction; MeOH: Methanol; MS: Mass spectrometry; MS/MS: Tandem mass spectrometry; MWCNTs: Multi-walled carbon nanotubes; NaOAc: Sodium acetate; NPD: Nitrogen phosphorous detector; SLE: Solid-liquid extraction; SPE: Solid-phase extraction; OCPs: Organochlorine pesticides; OPPs: Organophosphorus pesticides; PLE: Pressurized liquid extraction; PSA: Primary secondary amine; PYPs: Pyrethroid pesticides; RSD: relative standard deviation; UHPLC: Ultra high-performance liquid chromatography; USE: Ultrasonic solvent extraction; UV: Ultraviolet.

## Data Availability

Not applicable.
